# Community mental health care network: an evaluative approach in a Brazilian state

**DOI:** 10.1186/s13033-023-00578-7

**Published:** 2023-04-19

**Authors:** Vívian Andrade Araújo Coelho, Carlos Alberto Pegolo da Gama, Leonardo Isolani e Andrade, Mariana Arantes e Silva, Denise Alves Guimarães, Eliete Albano de Azevedo Guimarães, Celina Maria Modena

**Affiliations:** 1 Graduate Program in Public Health, René Rachou Institute/Fiocruz Minas, Belo Horizonte, Minas Gerais Brazil; 2 Department of Family Medicine, Mental and Collective Health (DEMSC), Medical School, Federal University of Ouro Preto (UFOP), R. Dois - Campus Morro do Cruzeiro, Ouro Preto, MG 35400-000 Brazil; 3 Federal University of São João Del Rei, Mid-West Campus (UFSJ/CCO), Divinópolis, Minas Gerais Brazil; 4 Graduate Program in Public Health, René Rachou Institute/Fiocruz Minas, Belo Horizonte, Minas Gerais Brazil

**Keywords:** Mental health, Community mental health services, Health services research, Health care quality, Access and evaluation

## Abstract

In recent decades, public policies of the Unified Health System (SUS) in Brazil have structured a community mental health care network (RAPS) based on various community actions and services. This study carried out evaluative research on the implementation of the structure and process dimensions of this care network in Minas Gerais, the second most populous state of Brazil, generating indicators that can enhance the strategic management of the public health system in the strengthening the psychosocial care in the state. The application of a multidimensional instrument, previously validated (IMAI-RAPS), in 795 of the 853 municipalities in Minas Gerais was carried out between June and August 2020. Regarding the structural dimension, we noticed an adequate implementation of services like ‘Family Health Strategy,’ ‘Expanded Family Health Center,’ and ‘Psychosocial Care Centers’ but a lack of ‘Beds in General Hospitals’ destinated to mental health care, ‘Unified Electronic Medical Records’ and ‘Mental Health Training Activities for Professionals.’ In the process dimension, adequate implementation of actions such as ‘Multidisciplinary and Joint Care,’ ‘Assistance to Common Mental Disorders by Primary Health Care,’ ‘Management of Psychiatric Crises in Psychosocial Care Centers,’ ‘Offer of Health Promotion Actions,’ and ‘Discussion of Cases by Mental Health Teams’ point to a form of work consistent with the guidelines. However, we detected difficulties in the implementation of ‘Psychosocial Rehabilitation Actions,’ ‘Productive Inclusion,’ ‘User Protagonism,’ ‘Network Integration,’ and practical activities for the effectiveness of collaborative care. We found a better implementation of the mental health care network in more populous, demographically dense, and socioeconomically developed cities, which shows the importance of regional sharing of services that are not possible for small cities. The evaluation practices of mental health care networks are scarce throughout the Brazilian territory, a fact also found in Minas Gerais, highlighting the need for its expansion not only in the scientific sphere but also in the daily life of the various levels of management.

## Introduction

In Brazil, a National Mental Health Policy was driven by social movements in the 1980s and became official in 2001 through Law 10.216 [1]. The restructured healthcare model was based on prior work carried out in countries with larger resources, and it aimed to shift the focus from hospital care to care in the community and primary healthcare services, placing the user and the protection of their human rights at the heart of its mission [2]. Since then, several changes based on the provision of psychosocial care have occurred relating to the setting up of services and clinical practices in the different regions, leading the Brazilian deinstitutionalization experience to have a prominent place in the field of global mental health [3, 4].

At that time, the Unified Health System (SUS) also took steps to regionalize care by defining decentralized responsibilities and joint planning, management, and financing instruments by municipal, state, and federal instances to reduce fragmentation and overcome the inequalities in the country [5]. This organizational arrangement has recently been strengthened by the healthcare network structuring, which brings together actions and services of varying complexity [6]. In 2011, the Psychosocial Care Network was defined as one of the priority areas for integrating mental health into the various points of care within the public health system, namely the primary healthcare services, specialized psychosocial care, urgent and emergency care, transitional residential care, hospital care, and strategies of deinstitutionalization and psychosocial rehabilitation [7].

This process has been reinforced in recent years, although data on monitoring and evaluation is still scarce. Until 2015, the Ministry of Health periodically published the "Mental Health in Data” bulletin [8]. However, government data on the topic has been limited since that date. In 2017, after a change in the direction of the federal government’s policies, recommendations were made to change the National Mental Health Policy [9]. Such a scenario broke a tradition of going along with social instances previously involved in its construction and proposing changes reminiscent of the past, with a return to specialization and centralization in the biomedical treatment model [1, 10], going against international tendencies [2, 11]. This reinforces the need for critical studies on the changes to mental health services in Brazil. Currently, the evaluation of the Brazilian mental health care network falls short of the expansion of the community care implemented, and most of the research carried out still focuses on the analysis of isolated services or specific populations. Moreover, few studies have used quantitative methodologies or validated and reproducible instruments for network evaluation, nor do they evaluate the actions performed daily for the provision of satisfactory care [12–15]. This lack of data weakens analysis of the public policy of the care model, leading to the risk that it might be dismantled under the auspices of little scientific evidence to maintain it [16].

This study therefore evaluates the structure and process [17] of mental health care network implementation in the municipalities of Minas Gerais. We aim to identify the strengths and weaknesses of its implementation and to produce scientific evidence to support the Brazilian public policy of improving mental healthcare provision.

## Methodology

This evaluative research focuses on analyzing the implementation of programs by studying the relationships between an intervention and its context during its implementation [17]. We studied the structure and process of RAPS implementation in Minas Gerais. We understand ‘structure’ as the relatively stable characteristics of the program, instruments, and resources, as well as the physical and organizational conditions; by ‘process,’ we mean the activities developed and the way the network operates [18].

A cross-sectional study was conducted involving persons responsible for performing actions relating to mental health in the state’s municipalities. Minas Gerais is Brazil’s second most populous state (21,040,662 inhabitants), with the fourth largest territorial area (557.448.8 km^2^). It is made up of 853 municipalities, 78.4% of which have fewer than 20,000 inhabitants and 91.7% of which have fewer than 50,000 inhabitants [19]. For the purposes of managing healthcare-related actions, the state is divided into 14 macro-regions, which in turn are divided into 89 health regions. Territorial divisions take the region's demographic, socioeconomic, geographical, sanitary, and epidemiological characteristics into account [20].

All state municipalities were considered eligible for this evaluation. In order to select the main person responsible for mental health actions of each municipality, we worked with the mental health coordination section of the Minas Gerais State Health Department (SES/MG). They helped with the study and put the researchers in touch with the 28 regional technical contacts for mental health at the SES/MG. With the assistance of regional contacts, an active search was conducted to locate the respondents. The research team contacted them via telephone or by WhatsApp^®^ messages. When the municipality did not have a specific coordinator for mental health, the questionnaire was sent to the professional or manager with the greatest level of knowledge of this area at the municipal level. We emphasized the anonymity of the answers to the respondents to guarantee maximum reliability in the information provided.

Data were collected between June and August 2020 through the previously validated multidimensional instrument for evaluating the implementation of a Psychosocial Care Network (IMAI-RAPS) [21]. As the data were collected at the beginning of the Covid-19 pandemic, we emphasized to the respondents that the information should refer to the pre-pandemic period to avoid biases. The questionnaire was electronically forwarded through the GoogleForms® software to respondents. In order to access the questionnaire, the respondents first read the free and informed consent form and accepted to participate in the research.

The IMAI-RAPS contains 55 objective questions with structured answers on a Likert scale regarding the following variables: (1) professional and sociodemographic characteristics of the person responsible for mental health in each municipality; (2) questions about the structure and process dimensions of RAPS, divided into five components defined in the logical model of the program [21]: Minimum Units (Mental Health Care and Psychosocial Rehabilitation), Connectivity (Network Articulation), Integration (Governance and Care Management), Normativity (Mental Health Policy and Social Participation and Control), and Structure (Implemented Services, Logistics System, and Health Education).

To analyze the structural dimension, in addition to the issues present in the IMAI-RAPS, we collected data regarding some key services of the mental health care network (Family Health Strategy, Expanded Family Health Center, Psychosocial Care Center, and Psychosocial Beds in General Hospitals)^1^ deployed in the state, using the DATASUS and e-GESTORab free access government databases as sources. These data were structured in indicators detailing the populational coverage, according to the guidelines of the Ministry of Health as described in a previous study [22].

The degree of implementation (DI) of the network in each municipality was defined through a scoring system developed by consensus between the researchers and mental health workers who took part in the research (stakeholders: members of the National Coordination of Mental Health, State Coordination of Mental Health of Minas Gerais, Municipal Coordination of Mental Health of Belo Horizonte—the capital of Minas Gerais, regional technical references of Minas Gerais State Health Department and members of the academic community).

This scoring system assigns a different weight to each criterion of IMAI-RAPS [21, 23]. For the calculation, we determined the observed values (Σ criteria points) and the DI (Σ observed/Σ of maximum expected points × 100) for each component. The total DI was obtained by summing all of the components. The scores obtained from the sum of the points regarding the criteria of each dimension were transformed into percentages regarding the maximum possible score. The DI therefore expresses a percentage of the recommended value for each criterion in the guidelines, with categories defined as follows: 75% to 100%, adequate implementation; 50% to 75%, partially adequate implementation; 25% to 50%, inadequate implementation; up to 25%, critical implementation.

Regarding the variables which may influence the implementation of the mental health care network, the following were considered: (1) population size of the municipalities (less than 20,000 inhab.; 20,000 to 50,000 inhab.; 50,000 to 99,999 inhab.; 100,000 to 299,999 1inhab.; 300,000 to 499,999 inhab.; over 500,000 inhab.), (2) municipal demographic density, and (3) the ‘Minas Gerais Social Responsibility Index’ (IMRS). The IMRS is a triennial index that aggregates information related to health, education, social vulnerability, public security, sanitation and environment, culture and sports of each municipality in Minas Gerais. The most recent IMRS used in this study was performed with data obtained in 2017, 2018, and 2019 [24]. These variables were chosen on the basis that municipalities with smaller population sizes could have a lower degree of implementation of the mental health care network due to a lack of management capacity and resources, both material and human. In terms of population density, Minas Gerais has municipalities that highly differ in territorial extension, demographic density, and socioeconomic characteristics. Thus, many municipalities in the state have a large rural territorial extension and small population, and health services may be at large distances; this may result in logistical difficulties and a lack of qualified human resources [25]. Regarding the IMRS, we sought a robust index that could numerically express the socioeconomic and health situation of each municipality in the state. We hypothesized that municipalities with higher IMRS have a higher degree of implementation of the mental health care network since their results for these other parameters are better.

Statistical analysis was performed in the Statistical Package for Social Sciences (SPSS)^®^. The Chi-Square or Fisher’s Exact test was used for analyzing the relationships between qualitative variables. In order to analyze the correlations between continuous variables, we used Spearman’s Correlation Test. The Kruskal–Wallis nonparametric test was used to analyze and compare the variables.

The study was approved by the Ethics Committee for research involving humans (CAAE: 77798217.1.0000.5545). It was funded by the Minas Gerais Research Support Foundation (FAPEMIG) and is part of the first author’s doctoral research. The anonymity of the respondents was guaranteed throughout the process.

## Results

Of the 853 health professionals invited to participate in the study, 795 answered the IMAI-RAPS. In the preliminary analysis, 10 municipalities were excluded from the sample because their answers were inconsistent with each other. Therefore, the answers provided by 785 responders were used in the statistical analysis, which is a representative sample of all municipalities in the state (92%; p < 0.01). The average age of the respondents was 39 years; they had spent an average of nine years working at SUS and six in mental health. Females made up 81.3% of respondents; in relation to the position held by the respondent in the municipal administration, 48.3% held the position of municipal technical reference for mental health, 19.7% were municipal coordinators of the mental health care, 14.4% were mental health care network professionals, 11.7% were coordinators from another health area, 4.6% were municipal health professionals, and 1.3% were coordinators of mental health specialized services. Regarding professional training, most participants (54.3%) were psychologists, 28.5% were nurses, 8% were social workers, and the others ranged from occupational therapists, nursing technicians, physicians, pharmacists, and physiotherapists, including a few workers from other areas of training. 67.8% of professionals claimed to have some sort of graduate qualification, with 18.9% having completed a specialization in mental health.

The analysis demonstrated that taking all the parameters into account, the degree of implementation of the mental health care network in Minas Gerais was ‘adequate’ in 22.2% of municipalities, ‘partially adequate’ in 60.6%, ‘inadequate’ in 15.9%, and ‘critical’ in 1.3%.

Regarding the structural dimension, Table 1 shows that state implementation was ‘partially adequate’ (67.3%). However, great variability was found in the degree of implementation of its components. A point to note is that, despite the ‘Component Services’ section of the mental health care network having a degree of implementation of 77.6%, the implementation of ‘Psychosocial Beds in General Hospitals’ (43.15%) was still low. Regarding the ‘Logistics System’ (58.3%), inadequate implementation of a unified electronic medical record system between the different mental health care network services in each municipality is noteworthy. The ‘Health Education’ component (38.4%) was inadequately implemented for all the evaluated criteria.

**Table 1:** Degree of implementation (DI) of the components and evaluation criteria of the structural dimension of community mental health care network (RAPS), Minas Gerais, Brazil, 2020

Dimension	Component	Evaluative criterion	Maximum points	Degree of implementation (DI)^a^ %
**Structure DI**^**a**^** 67.3%**	*Component Services* *of the RAPS* *DI 77.6%*	Family health strategy (iESF)^b^	20	**80.15**
		Expanded family health center (iNASF)^c^	20	**95.91**
		Psychosocial Beds in General Hospitals (iLHG)^d^	20	**43.15**
		Psychosocial Care Center (iCAPS)^e^	20	**144**
	*Logistics System* *DI 58.3%*	Availability of vehicle or equivalent (e.g., transportation voucher) to get users to the services	06	**66.31**
		Availability of vehicle for transportation of on-duty professionals	06	**74.20**
		Existence of unified electronic medical records for municipal health services	06	**34.27**
	*Health Education* *DI 38.4%*	Provision by the municipality of educational activities in mental health for mental health care network professionals	08	**35.00**
		Municipal incentive for the participation of mental health care network professionals in educational activities in mental health	08	**40.89**
		Consideration of previous work experience in mental health or the existence of appropriate training in the selection of professionals to work in mental health in the city	08	**36.78**

Values for the degree of implantation have been placed in bold only to stand out in the table

^a^Degree of implementation = Σ observed/Σ of expected maximum points × 100

^b^ iESF = covered population/total population

^c^iNASF = (NASF 1 × 31.050) + (NASF 2 × 13.800) + (NASF 3 × 6.900)/population

^d^iLHG = n^o^ of beds × 23.000/population

^e^ iCAPS = 100.000  ×  (CAPS 1 × 0.5) + CAPS 2 + (CAPS3 × 1.5) + CAPSi + CAPSad + (CAPSad 3 × 1.5)/population

^b,c,d^ For further information about the calculation of these parameters, see Coelho VAA, Andrade LI, Guimarães DA, Pereira LSM, Modena CM, Guimarães EAA, et al. Regionalization of psychosocial care: a panoramic view of the Psychosocial Care Network of Minas Gerais state, Brazil. Cien Saude Colet 2022; 27 (5): 1895–1909

Table 2 details the degree of implementation of the processes, which had an average score throughout the state of 60.3%. In the ‘Minimum Units’ component, the subcomponent ‘Mental Health Care’ (66.7%) is better implemented than the subcomponent ‘Psychosocial Rehabilitation’ (48.5%) in the evaluated state. The best-scored activities were ‘Multidisciplinary and Shared Care’ (68.2%) and ‘Conversation Circles for Health Promotion, Self-care, Adequate Use of Medications and/or Prevention of Abuse of Alcohol and Other Drugs’ (70.5%). The actions with the worst degree of implementation were ‘Deinstitutionalization’ (36.0%) and ‘Inclusion of Users in the Labor Market’ (31.3%). Regarding the ‘Connectivity’ component, we found that the ‘Case Discussion by the User Monitoring Team’ activities (74.4%) and the presence of ‘Collaborative care actions in the municipal mental health care network’ (74.8%) had the highest scores. However, when the evaluation criteria detailing collaborative care actions were analyzed, we observed that the ‘Inclusion of Case Discussions or Joint Casework’ (59.8%) and the ‘Weekly Attendance of Collaborative Care Professionals to the Units’ (37.8%) scored lower. Additionally, ‘Network Meetings’ (41.0%), an action also essential to collaborative care, were inadequately implemented. In the ‘Integration’ component, 46.3% of the municipalities in the state have specific coordination for mental health actions. Regarding ‘Establishment of Regional Mental Health Care Network,’ despite being well implemented (78.2%), the respondents stated that most are ineffective for comprehensive care in the health region (45.5%). In the ‘Normativity’ component, the subcomponent ‘Mental Health Policy’ had a degree of implementation of 63.5%. Even so, the activity ‘Evaluation and Monitoring of the Municipal Mental Health Care Network’ (35.7%) has not been properly implemented. The subcomponent ‘Participation and Social Control’ (46.9%) also showed inadequate implementation, and the ‘User Assembly’ activity had a critical implementation (20.7%).

**Table 2:** Degree of implementation (DI) of components, subcomponents, and evaluative criteria of the process dimension of community mental health care network (RAPS), Minas Gerais, Brazil, 2020

Component	Subcomponent	Activity	Evaluative criterion	Maximum points	Degree of implementation (DI) %
Minimum units **DI 62.9%**	Mental Health Care **DI 66.7%**	Multidisciplinary and shared care **DI 68.2%**	Multidisciplinary care with shared decisions in municipal mental health care	10	**74.1**
			Participation of the psychiatrist (municipal or from other agreed municipalities) in the discussion of the case	06	**50.9**
			Comprehensive case care of users with common mental disorders by primary healthcare services	10	**77.2**
			Comprehensive case care of users with severe mental disorders by primary healthcare services	10	**69.5**
			Comprehensive case care of users in psychological distress secondary to the use of alcohol or other drugs by primary healthcare services	10	**64.2**
			Assistance to children and adolescents with psychological distress in municipal services	10	**66.3**
		Therapeutic workshops and/or expressive or physical group activities **DI 61.7%**	Presence of therapeutic workshops and/or expressive or physical group activities in municipal mental health care	10	**61.7**
		Elaboration of the singular therapeutic project **DI 61.7%**	Elaboration of the singular therapeutic project	08	**61.7**
		Crisis management **DI 65.3%**	Crisis care in municipal or neighboring agreed-on Psychosocial Care Centers in the region	10	**70.4**
			Crisis management without referral to the psychiatric hospital	10	**56.5**
			Assistance to severe cases of psychic suffering secondary to the use of alcohol or other drugs in municipal or neighboring agreed-on Psychosocial Care Centers in the region	10	**68.9**
		Conversation circles and/or other approaches to health promotion, self-care, appropriate use of medications, and/or prevention of alcohol or other drug abuse **DI 70.5%**	Offer of conversation circles, groups, workshops, or other actions on health promotion	06	**82.4**
			Strategies for preventing the harmful use of alcohol or other drugs	08	**61.5**
	Psychosocial rehabilitation **DI 48.6%**	Promotion of leisure alternatives such as sports and cultural activities DI 65.2%	Promotion of sports and/or cultural activities for the population by the municipality	06	**65.2**
		Deinstitutionalization **DI 36.1%**	Actions for deinstitutionalization of long-term users in psychiatric hospitals or other institutions	08	**36.1**
		Psychosocial interventions **DI 55.1%**	Psychosocial interventions for user integration into community support networks	10	**55.1**
		Actions to increase the autonomy of users **DI 52.3%**	Actions to increase the autonomy of users	10	**57.3**
		Inclusion of users in the labor market **DI 31.3%**	Actions to insert the user into the labor market	08	**31.3**
Connectivity **DI 61.7%**	Network articulation **DI 61.7%**	Team meetings, network meetings, matrix support of primary care teams, of the urgency and emergency networks, and reference hospital services **DI 61.5%**	Case discussions among the team members	10	**74.4**
			Case discussions between teams of different services	10	**62.7**
			Connection of actions between the different services	10	**57.2**
			Collaborative care actions in mental health at municipal mental health care network	10	**74.8**
			Inclusion of case discussions or joint casework in collaborative care actions	10	**59.8**
			Weekly attendance of collaborative care professionals at the units	06	**37.8**
			Case discussions between the team accompanying the user and the hospital team when users are hospitalized (for reasons related to mental health)	08	**51.3**
			Network meetings (involving professionals from various services of the municipal mental health care network)	10	**41.0**
		Development of actions in conjunction with other sectors of the Public Administration for case management or health promotion (e.g., justice, leisure, education, sports, social work) DI 60.8%	Development of actions in conjunction with other sectors of the municipal administration	10	**60.8**
Integration **DI 61.7%**	Governance **DI 60.0%**	Creation of specific spaces for mental health management at the municipal, regional, state, and federal levels **DI 46.2%**	Presence of a specific coordinator for mental health at the municipal level	08	**46.2**
		Establishment of Regional RAPS **DI 64.0%**	Agreements between regional municipalities for the sharing of mental health care network services	10	**78.2**
			If yes to the previous question: are the pacts in which the municipality participates adequate for the mental health care users?	08	**45.5**
	Care Management **DI 62.2%**	Construction of flows between the various RAPS services **DI 55.4%**	Existence of structured flows for the circulation of users through the various health services of the local mental health care network	08	**55.4**
		Responsibility of one or more professionals by the user along their path with RAPS **DI 64.3%**	Assignment of one or more professionals to be responsible for each user during their course through the mental health care network	08	**68.5**
			Existence of discussion among professionals to articulate a joint care project in the transition between services	06	**57.3**
		Co-responsibility for the user among the professionals of different services of RAPS **DI 58.6%**	Existence of co-responsibility of professionals from different services in the follow-up of the case	06	**58.6**
Normativity **DI 54.8%**	Mental Health Policy **DI 63.5%**	Knowledge and acceptance of government guidelines, protocols, and instructions for mental healthcare by the correspondent professionals **DI 77.6%**	Basis of mental health actions in the municipality in the guidelines of the National Mental Health Policy	10	**81.8**
			Knowledge by the municipality's mental health professionals about how the mental health care network operates	06	**68.9**
		Evaluation and monitoring **DI 35.7%**	Actions for evaluation and monitoring of mental health care at the municipal leve	08	**35.7**
	Participation and social control **DI 47.0%**	Assembly of users **DI 20.8%**	Existence of user assemblies in municipal mental health services	08	**20.8**
		Approaches that put the expressed needs of users at the heart of their care **DI 65.4%**	Participation of users and/or their families in decisions about their therapeutic projects	08	**65.4**
		Participation of professionals, users, and family members in collegiate, commissions, forums, or other spaces for collective discussions on mental health **DI 46.4%**	Participation of users and family members in spaces of collective discussion in mental health	06	**35.6**
			Participation of municipal mental health care network professionals in spaces of collective discussion about mental health	06	**53.3**

Values for the degree of implantation have been placed in bold only to stand out in the table

Regarding the association of the degree of implementation of the mental health care network in the municipalities of Minas Gerais with variables of external to the network, we found a significant and positive correlation between the degree of implementation and the analyzed data (Table 3), as follows: population size (p-value 0.002; r = 0.235), demographic density (p-value 0.015; r = 0.121), and IMRS (p-value 0.015; r = 0.136).

**Table 3:** Association of the degree of implementation (DI) of community mental health care network (RAPS) with the variables of the external context, Minas Gerais, Brazil, 2020

External Context		Average DI [95% CI]	Median DI	Standard deviation	P-value (b)	Correlation (c)
Municipalities Population (inhab.)	General DI	0.63 [0.62–0.64]	0.65	0.15	–	–
	< 20.000	0.61 [0.60–0.62]	0.62	0.15	0.002	0.235 (0.000)
	20 to 50.000	0.69 [0.66–0.71]	0.71	0.14		
	50 to 99.999	0.69 [0.65–0.73]	0.70	0.14		
	100 to 299.999	0.72 [0.67–0.76]	0.74	0.10		
	300 to 499.999	0.72 [0.53–0.90]	0.73	0.18		
	> 500.000	0.72 [0.57–0.88]	0.69	0.12		
Population Density		69.29 [45.75–92.82]	22.85	335.92	0.015	0.121 (0.001)
IMRS		0.60 [0.59–0.60]	0.59	0.06	0.015	0.136 (0.000)

^a^Degree of implementation (DI) = Σ observed/Σ of expected maximum points X 100);

^b^Pearson’s Chi-Square test with a significance level of 95%;

^c^Spearman’s test

Approximately 92% of the state’s population resides in municipalities with a degree of implementation of the mental health care network classified as adequate or partially adequate. In addition, all municipalities were evaluated as having critical implementation, and most of those with inadequate implementation has a population of < 50,000 inhabitants. We also found that the municipalities that have a Psychosocial Care Center—or make use of an agreed neighboring Psychosocial Care Center—have significantly higher values in relation to the degree of implementation (Table 4).

**Table 4:** Distribution of the Minas Gerais municipalities through the degree of implementation (DI) Association between population size, use of Psychosocial Care Center (CAPS), and degree of implantation (DI) of community mental health care network (RAPS) in municipalities of Minas Gerais, Brazil, 2020

		Average		Adequate		Partially adequate		Inadequate		Critical		p-value^b^	Correlation^c^
				Mun	Pop	Mun	Pop	Mun	Pop	Mun	Pop		
Population Size	< 20,000	0.61		111	988296	382	2951939	112	814915	8	67,603	0.002	0.235 (p = 0.000)
	20 to 50,000	0.69		38	1224912	57	1685871	8	195886	2	44,625		
	50 to 99999	0.69		12	863282	22	1596282	4	284151	0	0		
	100 to 299999	0.72		10	1628443	11	1575524	1	150833	0	0		
	300 to 499999	0.72		2	763620	2	735165	0	0	0	0		
	> 500000	0.72		1	2501576	2	1223380	0	0	0	0		
Does the municipality have CAPS?	Yes, municipal	0.71		98	7223317	147	7273249	14	581118	0	0	0.000	–
	No, but users use the service of another agreed municipality	0.64		56	501276	185	1259903	40	268040	0	0		
	No	0.55		19	241860	136	1184509	66	568362	8	101218		
	Does not apply	0.59		0	0	2	22,856	0	0	0	0		
	Unknown	0.49		1	3676	6	27,644	5	28,265	2	11,010		
Total		N	0.63	174	7970.129	476	9768161	125	1445785	10	112228	–	–
		%		22.17%	41.30%	60.64%	50.62%	15.92%	7.49%	1.27%	0.58%		

^a^Degree of implementation (DI) = Σ observed/ Σ of expected maximum points X 100)

^b^Pearson's Chi-Square test with a significance level of 95%

^c^ Spearman's test; *Mun* municipality, *Pop* population

## Discussion

An evaluative survey of the implementation of mental health care networks was carried out in a representative sample of all municipalities in Minas Gerais. The areas studied allow for a detailed analysis of the structure and specific activities that constitute the complex range of actions necessary for the satisfactory provision of psychosocial community care [2]. We were able to demonstrate which advances have taken place in psychosocial care in Minas Gerais, as well as any limitations that may compromise care and the deinstitutionalization process.

An adequate implementation of services (e.g., Family Health Estrategy (ESF), Expanded Family Health Center (NASF), and Psychosocial Care Center (CAPS)) and actions, such as multidisciplinary and joint casework in mental health care; assistance for common mental disorders by primary health care teams; management of psychiatric crises in Psychosocial Care Center; offer of health promotion actions; case discussions by teams; and basing municipal actions in the National Mental Health Policy, stand for the consolidation of working in line with the national guidelines in the state of MG [9, 11].

However, despite the advances observed in care actions based on an amplified clinic, community mental health care providers should focus not only on the management of mental health symptoms but also on developing users’ capacities, allowing them to be involved in their care actively and validating their aspirations to maximize their quality of life [2, 26]. We detected difficulties in the mental health care network of Minas Gerais with regard to the implementation of ‘Psychosocial Rehabilitation Actions,’ ‘Psychosocial Interventions,’ ‘Productive Inclusion,’ and ‘Users´ Protagonism.’

This finding is consistent with those found in other Brazilian regions [13, 27–29] and international reports [2, 30], reflecting the challenges of changing a logic limited to assistance to a users’ emancipatory scenario, which is necessary for community care in mental health [2, 26, 31]. In this sense, a paradigm change would require changes in how health professionals work, making it possible to break with the positivist principles and traditional psychiatry and find new ways of providing psychosocial care. These changes would need to surpass the traditional healthcare limit by mobilizing community resources in the territory to establish relationships between citizens and city life [26, 27]. Brazil has been placing the user at the center of the health care system, using several initiatives to reinvent citizenship, such as users and familiar associations, social cooperatives and solidarity economy groups, art and culture projects, participation in policy-making forums and Health Councils, and many other ways of promoting human rights and cultural diversity [26]. Nonetheless, the analysis of the implementation of the evaluation criteria contained in the subcomponents ‘Psychosocial Rehabilitation’ and ‘Social Participation and Control’ exemplify how these practices are still scarce in the mental health care network in Minas Gerais. Strategies to prevent the use of alcohol and other drugs also need to be improved, such as promoting sports and cultural activities by municipalities.

Moreover, recent qualitative research analyzing the points of view of mental health care network users [31, 32] demonstrated that care processes are still marked by discrimination and limited social participation on a national scale. Mental health care networks and other social services are used when pathologies and the need for guardianship arise. These studies also demonstrate challenges in implementing mental health actions in primary health care and other non-specialized sections of the mental health network, in addition to the fragility in articulating and implementing integrated care in all services, keeping demands on mental health centralized in specialized services. We also found that the follow-up of users with severe mental disorders or secondary to the use of alcohol or other drugs by primary health care is still below the desired level in the state analyzed. A higher degree of implementation score for municipalities that have Psychosocial Care Centers, or those which are able to use the services of a neighboring municipality, may indicate that this service is an articulator in mental health care, as well as a challenge in incorporating psychosocial care by other mental health care network services.

Similarly, even though the ‘Definition of one (or more) reference professional (s) responsible for the user throughout their follow-up’ and the ‘Elaboration of the singular therapeutic project’ were evaluated as being partially implemented, the degree of implementation was lower when analyzing the ‘Connection of actions between different services,’ the ‘Existence of structured flows for the circulation of users through the various health services of the local mental health care network,’ the ‘Existence of discussion among professionals for the articulation of a joint case project in the transition between the mental health care network services,’ and the ‘Existence of co-responsibility among professionals from different services in the follow-up of the case.’ This demonstrates that coordination of care is one of the great challenges of the institution of health care networks in Brazil and other countries [33]. In decentralized public health systems, as in the Brazilian case, areas of concern include limited communication and exchange of clinical data among professionals, access barriers in referrals, and differences in treatment among professionals of distinct services [34]. With regard to the mental health care network, this process can be even more complex, as the structuring of an integrated network responsible for a range of areas, going from the care of mild mental disorders to crisis management, as well as deinstitutionalization and psychosocial rehabilitation, presupposes the development of refined coordination, regulation, and planning tools [35].

To overcome such problems, the *collaborative care* model, as proposed by the support matrix in Brazil, has been shown in previous studies to enhance not only the integration and coordination of care in healthcare networks but also the training of professionals [16]. This proposal, consistent with a network structure, aims to provide a new way of organizing healthcare provision, with workflows involving different teams in providing user care, with joint responsibility for cases, integrating different specialties and levels of care [33, 36, 37]. The present results show an adequate implementation of the evaluative criterion ‘Collaborative care actions for mental health in municipal mental health care network.’ However, when analyzing in detail the actions that would be expected in this proposal (‘Inclusion of case discussions or joint care in collaborative care actions,’ ‘Weekly attendance of the collaborative care professionals in the matrix unit,’ ‘Network meetings”, ‘Existence of discussion among professionals for the articulation of a joint case project in the transition between services,’ among others), we found a lower implementation level. This may suggest that practical difficulties exist in implementing this care model in the mental health care network of Minas Gerais, as is the case in other Brazilian locations [36]. Our findings showed that discussions between professionals (“Case discussions by the team that accompanies the user”) occur within the scope of mental health specialized teams. Therefore, they do not include other sections of the network that, precisely because they are not specialized, are the ones that are in most need of actions to enhance their capacity to act in mental health through learning spaces and work. A low level of encouragement for professionals to participate in mental health training activities and a lack of priority for hiring professionals with previous experience or training in the area to work in mental health care network was also noted in this study. These results suggest that professionals may have few resources to deal with highly complex problems, such as those that usually require care in mental health care [36]. In addition to these challenges, we found that few municipalities have a unified electronic medical record for health services, which would allow better coordination of information within the network [38].

A low participation of psychiatrists in the discussions and decision-making process was also shown. In other national and international situations [39], only a minority of specialized physicians also perceive themselves as jointly responsible for patients during their trajectory through different levels of care and/or participate in anything other than individual medical consultation. The international relevance of this issue prompted the editors of the leading medical journal *The Lancet*, in a recent publication [11], to encourage physicians and governments to do more than just prescribe psychotropic drugs to address mental disorders. They argue that an integrative and holistic approach that would more broadly account for the social determinants of mental illness to advance the field of psychiatry is required since, as previously emphasized, classical treatments, including medications and oral therapy, have limitations in the mental health clinic. The very existence of a specific question regarding psychiatrists in the questionnaire used in this study demonstrates a concept of treatment that is still centered around the physician. Previous research has shown that this is still an issue in Minas Gerais’s mental health care network and needs to be quantified [40, 41]. Nevertheless, in order to achieve the necessary engagement of health professionals (physicians and others) in the new mental health processes, it may be necessary to address factors such as the overvaluation of individual consultations to the detriment of spaces for joint discussion to conduct the case; the lack of clarity about strategies for the practice of collaborative care, case coordination, and longitudinal follow-up; the lack of knowledge and/or skills and/or specific training for the development of the work to be performed, lack of time due to work overload, and lack of interest and unsafe working conditions, which all lead professionals to see patient consultation as an isolated act [32, 34, 35, 39, 42].

Regarding crisis management, the degree of implementation of the evaluative criterion “Crisis Management Without Referral to the Psychiatric Hospital” was close to 50%, despite the coverage of Psychosocial Care Centers in Minas Gerais being higher than the level stipulated by the Ministry of Health (DI: 144%) and that respondents reported that crisis care is available in those services. It is important to highlight that the low degree of implementation of the ‘Deinstitutionalization’ criteria (36%) may refer to the extent of the process conducted, in the early 2000s, by the National Mental Health System on closing psychiatric long-stay hospitals and the limitation of short-term inpatient hospitalization. On this issue, previous research has shown a scarcity of Psychosocial Care Centers aimed at specific populations (CAPS AD-alcohol and drugs and CAPSi-children and adolescents) and Psychosocial Care Centers that operate 24 h a day. These studies also evidenced the insufficient availability of psychosocial beds in general hospitals for comprehensive community crises management in MG [22, 40, 43, 44] and other Brazilian states [28, 29, 45]. These data may also explain other difficulties found in our study regarding the incorporation of Minas Gerais’s mental health care network of clinics that work not just in suppressing symptoms but also encourage a deeper reflection on the subjective social, family, and relational aspects of crisis. Additionally, as highlighted by Martins (2017) [46], the lack of personnel training can compromise the management of crises, as these crises include symptoms that may be confused with strangeness and social disturbance. Such misconceptions pervade health professional circles, as does the ideal of normality still present in our society, once again demonstrating the challenges of transforming the social relationship with insanity and human differences.

The confirmation of our hypothesis that a mental health care network in more populous, demographically dense cities with higher IMRS have a better implementation score highlights the importance of regional structuring of the network and the sharing of services that are impractical for small rural cities, as they make up the vast majority of the municipalities of Minas Gerais. Despite this, fewer than half of respondents judged that existing regional service sharing is adequate for the comprehensive mental health care of users. This difficulty in the regionalization of psychosocial care has already been reported in previous studies in MG [22, 40] and other states in the country [28, 29, 33]. It is possibly linked to insufficient resources and the difficulty of collaboration between municipalities, bureaucratic barriers to accessing federal resources for improving health regionalization, partisan political interests, a lack of qualifications and regional vision by the professionals responsible for these areas at the municipal level, in addition to complications of the state and federal government to plan and organize the coordinated management of regional networks. Furthermore, future studies must investigate if municipalities with large demographic densities, characterized by urban agglomerations and conurbations, should have more widely implemented actions but suffer overload due to the large population assisted.

As previously mentioned, there are few evaluative research on RAPS practices throughout the Brazilian territory, and the low implementation of the evaluation criterion ‘Actions for evaluation and monitoring of mental health care at the municipal level’ highlights the need to expand these practices not only in the scientific sphere but also in the daily practices at the different management levels within Minas Gerais.

The methodology used in this study was chosen to carry out a primary assessment in Minas Gerais. However, we could not understand all the complexity and tensions involved in developing expanded care in mental health. Elements such as the composition of the multidisciplinary technical team in each municipality, working conditions, professional turnover, the physical structure of services, mapping of other services within the network (living centers, therapeutic residences, reception units, among others), and inclusion of users and other network professionals in the evaluation should be considered in subsequent investigations. It is also important to mention that, since most of the respondents chosen in this study are mental health professionals, their answers reflect their point of view and should be complemented later with research analyzing the view of professionals from other RAPS services (e.g., Primary Health Care professionals, general hospital workers, among others) in relation to the evaluated criteria. However, the value of an evaluative analysis and the use of a validated instrument for the analysis of RAPS in a representative sample of the municipalities of Brazil's second most populous state stands out.

## Final considerations

As cited by World Health Organization (WHO) [4], the Brazilian mental health care network illustrates how a nation can establish health services on a large scale rooted in the principles of human rights and recovery. The comprehensive services network under the SUS results from the significant psychiatric reforms introduced in the 1980s. These reforms aimed to move the treatment focus from hospitals to communities in a supportive legal and regulatory environment. Nevertheless, it is imperative to carry out studies to evaluate the actions implemented after these changes were made to the mental health care model in the country.

The results of this evaluation show a distinguished implementation of a complex psychosocial clinic in the mental health care network of Minas Gerais, despite the recent economic and political crises combined with austerity policies that took place in recent years in Brazil [47] and are in line with the current international conjuncture [30]. As in most countries, challenges persist in the mental health care network in Minas Gerais, including limited professional training, trouble in developing policies aligned with the needs of the population and human rights conventions, limited social participation, and limited promotion and prevention strategies in mental health. We hope that the data generated here can aid the strategic management of SUS to provide comprehensive mental health care.

## Data Availability

Data sets used and/or analyzed during the current study may be made available by the corresponding author upon reasonable request.

## Footnotes

1 Family Health Strategy (ESF) is a program for the organization of primary health care in Brazil that prioritizes actions to promote, protect and recover health, in an integral and continuous way. It is considered the gateway to the health system and the organizer of the local health network. Together with the Psychosocial Care Centers (CAPS), the ESF is responsible for coordinating care for users in the psychosocial care network. Expanded Family Health Centers (NASF) are multidisciplinary teams with a wide range of specialists, including in mental health, who provide direct general support to ESF intending to increase it’s capacity to deal with the health problems of the territory. The psychosocial beds in the general hospital (LHG) were designed for the management of crises that go beyond the resolving capacity of the CAPS, avoiding hospitalization in psychiatric hospitals.
